# *SCN11A* gene deletion causes sensorineural hearing loss by impairing the ribbon synapses and auditory nerves

**DOI:** 10.1186/s12868-021-00613-8

**Published:** 2021-03-22

**Authors:** Mian Zu, Wei-Wei Guo, Tao Cong, Fei Ji, Shi-Li Zhang, Yue Zhang, Xin Song, Wei Sun, David Z. Z. He, Wei-Guo Shi, Shi-Ming Yang

**Affiliations:** 1 College of Otolaryngology Head and Neck Surgery, Chinese PLA General Hospital, Beijing, China; 2 National Clinical Research Center for Otolaryngologic Diseases, Beijing, China; 3 Key Lab of Hearing Science, Ministry of Education, Beijing, China; 4 Beijing Key Lab of Hearing Impairment for Prevention and Treatment, Beijing, China; 5 Clinical Hearing Center of Affiliated Hospital of Xuzhou Medical University, Xuzhou, Jiangsu China; 6 Department of Communicative Disorders and Sciences, Center for Hearing and Deafness, The State University of New York at Buffalo, Buffalo, NY USA; 7 Department of Biomedical Sciences, Creighton University School of Medicine, Omaha, NE 68178 USA; 8 State Key Laboratory of Toxicology and Medical Countermeasures, Beijing Institute of Pharmacology and Toxicology, Beijing, China

**Keywords:** TTX resistant sodium channels, Expression, Nav1.9 knockout, Ribbon synapse, SGN, Progressive hearing loss, Synaptopathy

## Abstract

**Background:**

The *SCN11A* gene, encoded Nav1.9 TTX resistant sodium channels, is a main effector in peripheral inflammation related pain in nociceptive neurons. The role of *SCN11A* gene in the auditory system has not been well characterized. We therefore examined the expression of *SCN11A* in the murine cochlea, the morphological and physiological features of Nav1.9 knockout (KO) ICR mice.

**Results:**

Nav1.9 expression was found in the primary afferent endings beneath the inner hair cells (IHCs). The relative quantitative expression of Nav1.9 mRNA in modiolus of wild-type (WT) mice remains unchanged from P0 to P60. The number of presynaptic CtBP2 puncta in Nav1.9 KO mice was significantly lower than WT. In addition, the number of SGNs in Nav1.9 KO mice was also less than WT in the basal turn, but not in the apical and middle turns. There was no lesion in the somas and stereocilia of hair cells in Nav1.9 KO mice. Furthermore, Nav1.9 KO mice showed higher and progressive elevated ABR threshold at 16 kHz, and a significant increase in CAP thresholds.

**Conclusions:**

These data suggest a role of Nav1.9 in regulating the function of ribbon synapses and the auditory nerves. The impairment induced by Nav1.9 gene deletion mimics the characters of cochlear synaptopathy.

## Background

The Nav1.9 sodium channel, encoded by *SCN11A* gene, was first identified in 1996 as an unusual voltage-gated sodium channel called SNS [1]. The Nav1.9 channel is well known for its presence in small-diameter nociceptive neurons dorsal root ganglion (DRG) [2, 3], trigeminal ganglia [4, 5] and myenteric intrinsic primary afferent neurons [6]. Recent studies found that Nav1.9 channel also expressed in photoreceptors and Muller glia in the visual system [7]. Nav1.9 shares only 50% identity with the other voltage-gated Na^+^ channel isoforms, but it doesn’t belong to a new Nav subfamily according to phylogeny [8]. Nav1.9 carries a serine (S) residue in the DI-SS2 pore region, rather than a tyrosine (Y) or a phenylalanine (F), which markedly reduces the affinity of the TTX-channel interaction by more than 200-fold [9].

Nav1.9 typically exhibits ultra-slow kinetics with an activation at around − 65 mV, lower than Nav1.1, a TTX sensitive sodium channel [10], the consolidation of which is important for the maturation of afferent fiber in the weeks after hearing onset. Unlike Nav1.1, Nav1.9 does not contribute so much to the amplitude of action potential, but facilitate excitation of small depolarization by amplifying receptor potentials. The ‘ultra-slow’ inactivation of Nav1.9 renders the persistence of the sodium current after activation, corresponding to a wide range of voltage to keep a persistently open channel close to the resting membrane potential [11]. The biophysical properties of Nav1.9 channels suggest their probable contribution to prolonging the response to a subthreshold stimulus and supporting repetitive firing [12].

Nav1.9 mediates tissue-damage in DRG with unmyelinated C fibers by transforming receptor potentials into action potentials. Nav1.9 KO mice shows less stimulation-induced calcitonin gene-related peptide (CGRP) release form skin, which implies that Nav1.9 has a role in modulating neurotransmitter release from afferent nerve endings [13]. Cochlea includes two types of auditory nerves. Type I afferents are myelinated innervating to IHCs, while Type II afferents are unmyelinated innervating outer hair cells (OHCs). Type II afferents may be the cochlearnociceptors, as highly enriched gene ontology (GO) terms in type II neurons (*Prph*+,* Th*+), were associated with “response to stress” and “pain” by single cell RNA-seq sequencing [14].

Multiple TTX-sensitive Na^+^ currents, including a subthreshold persistent Na^+^ current (I_NaP_), a resurgent Na^+^ current (I_NaR_) and fast inward sodium currents, could be recorded in cultured SGNs after the onset of hearing, while the function of Nav1.9 in auditory sensory system remains enigmatic [15, 16]. The gene expression microarray shows that, *SCN11a* shares a similar pattern with atonal homolog 1a (*Atoh1*) during hair cell differentiation, which is a well-known factor during inner ear development [17]. The relative expression level of *SCN11a* in cochlea is 1.7 compared with that of Atoh1 as 5.2 [18]. Moreover, Nav1.9 protein is expressed in cartwheel cells (CWCs) in the dorsal cochlear nucleus (DCN). And Nav1.9 is proved to contribute to respond with compound action potential (CAP) containing single action potentials (SAPs) superimposed on a slow depolarization [19].

In this paper we studied the expression and function Nav1.9 in the cochlea of mice. We found abnormal ABRs and CAPs, as well as decreased presynaptic CtBP2 puncta and SGN in Nav1.9 KO mice, suggesting impairment of the auditory signal transmission. Nav1.9 may contribute to auditory neurotransmission, sharing a similar protein expression pattern in nociceptive neurons of DRG as an effector of peripheral pain hypersensitivity.

## Methods

All animal procedures were carried out in accordance with the Policy on Human Care and Use of Laboratory Animals at PLA General Hospital and approved by the Institutional Animal Care and Use Committee (process no. 2018-X14-84).

### Construction of Nav1.9^−/−^ mice using CRISPR/Cas9

**Fig. 1:**
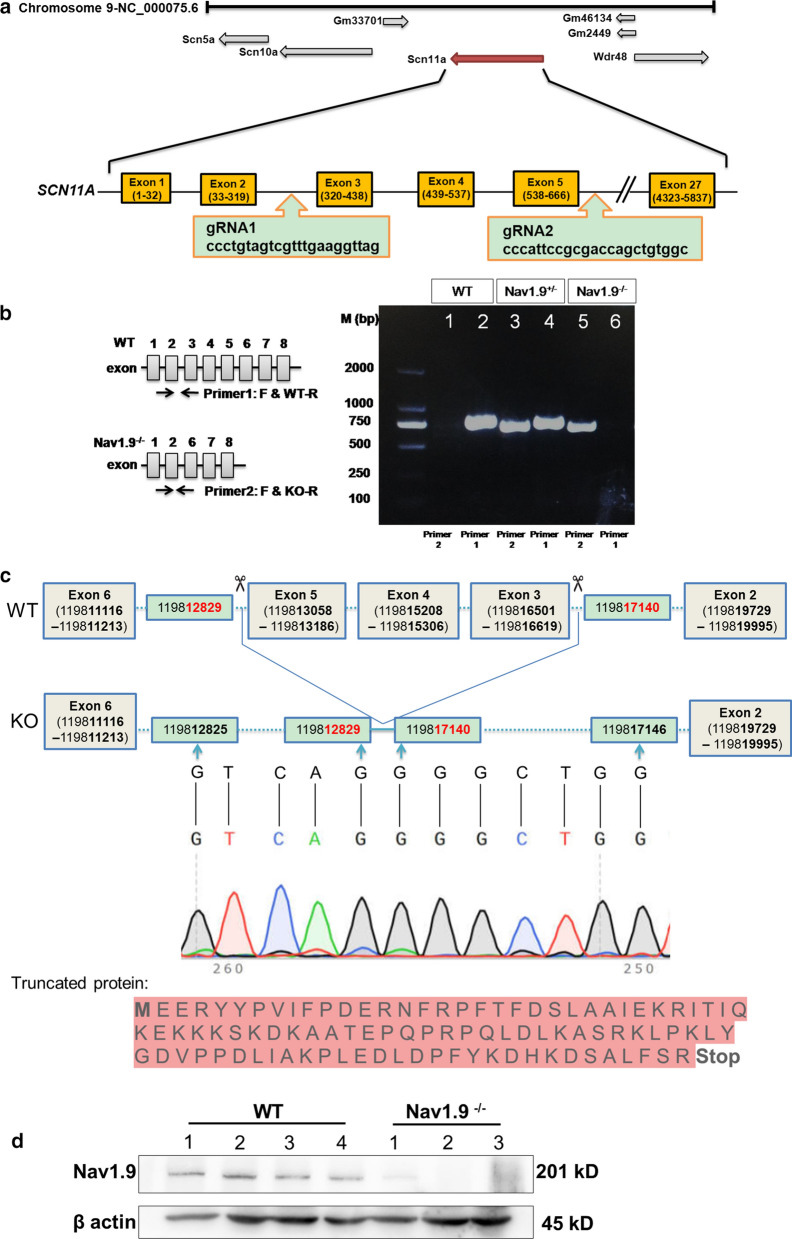
CRISPR/Cas 9-mediated generation of a Nav1.9^−/−^ mouse model. **a** A representative illustration of the CRISPR/Cas9 targeting strategy for generating Nav1.9 knockout (KO) mice. The Cas9 mRNA and two single guide RNAs targeting a region from SCN11A exon 3 to 5, were microinjected into mouse zygotes. **b** Schematic diagram of primer pair design for PCR genotyping, a representative PCR genotyping result for Nav1.9 wild-type (WT), homozygous (Nav1.9^−/−^) and heterozygous (Nav1.9^+/−^), the region of junction of DSB is absent in the WT mice. Primer 2: primer pairs containing forward primer and KO specific reverse primer; Primer 1: primer pairs containing forward primer and WT specific reverse primer. **c** This successfully eliminated all of exon 3, 4 and 5, as confirmed by Sanger sequencing, induces reading frame shift and thus a premature translational- termination codon during the truncated protein expression. **d** The protein expression of Nav1.9 in the cochleas of Nav1.9^−/−^ mice (n = 3) or WT mice (n = 4) was measured by western blot

Nav1.9^−/−^ ICR mice were obtained from CasGene Biotech. Co., Ltd. The specific single-guide RNAs (gRNAs) were designed and synthesized (gRNA1: ccctgtagtcgtttgaaggttag; gRNA2: cccattccgcgaccagctgtggc). To generate Nav1.9 knockout mice, gRNAs targeting a section of the *SCN11A* gene encoding sequence beside the PAM sequence, and Cas9 nucleases could introduce a double-strand break (DSB) (Fig. 1a). The all-in-one plasmid expressing Cas9 and gRNAs was microinjected into fertilized eggs under micromanipulation. About 3 weeks after the injection of eggs to a pseudopregnant female, the founder mice were obtained. The homologous genetic deletion was identified by PCR amplification (forward primer: 5′-GACACTCTGGCGGT GCCTTCC-3′; KO specific reverse primer: 5′-TTGCTCCCACCTTACCAATACAGACTC-3′; WT specific reverse primer: 5′-CGACATTCCTCCGAGACCTGTTAGA-3′) and DNA sequencing analysis. The off-target effect and germline transmission to the offspring were then determined. Heterozygous males and females were mated to produce wildtype (WT), heterozygous and homozygous offspring. Mice were maintained in a humidity and temperature-controlled IVC animal experiment system with 12 h light/dark cycle.

### Real-time quantitative PCR

Real-time quantitative PCR (qPCR) was used to identify mRNA expression of Nav1.9 in cochlea of mice at different time points after birth (P0, P7, P14, P21, P28 and P60), with five mice in each group. Total RNA from modiolus was prepared by RNeasy Mini Kit (cat. 74104, QIAGEN) followed by purity determination and quantitation. cDNA was synthesized by random primer using the TransScript first-strand synthesis supermix for RT-PCR (TransGen Biotech). qPCR with reaction volume of 20 μL containing primers (200 nM) and 1 μL cDNA, was performed on CFX96™ Real-Time PCR Detection Systems (Bio-Rad Life Science). Specific primers for Nav1.9, Nav1.1 and GAPDH (housekeeping gene) were designed and synthesized (Table 1). The following run protocol for amplification was used: denaturation (94 °C for 30 s), amplification and quantification by 45 cycles (94 °C for 5 s, 56.8 °C for 15 s, 72 °C for 10 s), melting curve (65–95 °C with a heating rate of 0.1 °C/s). Negative controls with Ct values more than 38 or no visible amplification curve were tested in each run. The relative expression level was calculated by the $2^{{ - \Delta \Delta {\text{Ct}}}}$ method as previously described [20, 21].

**Table 1:** Primers for real-time RT-PCR analysis

Target	Sequence (5′ to 3′)	
	Forward primer	Reverse primer
Nav1.1	TTCAGGGGCTATCGAGGC	TGCTGAATAATGAGTGTACCAAAAT
Nav1.9	GAAAAAGTTAGGTGGCCAAGACAT	GTTGGGCTGGCCTTCAGATT
GAPDH	GGAATGCCTACCTTGCCCTG	ATGTCTTGGCCACCTAACTTTT

### Cochlea sectioning and scanning electron microscopy (SEM)

After the anesthesia by an intraperitoneal injection of pentobarbital sodium (50 mg/kg), the P60-adult mice were sacrificed by decapitation. The cochleas were isolated and fixed in 4% paraformaldehyde overnight, followed by decalcification in 10% EDTA at room temperature for 24 h. For cochlea immunostaining, the tissues were cryoprotected successively in 20% and 30% sucrose in PBS for 2 h and in Tissue Freezing Medium (OCT) at 4 ℃ until they sank. The cryoprotected tissues were sectioned at 10 μm for immunostaining, while sections at 2 μm were prepared for hematoxylin and eosin staining.

For the SEM, the cochlea was perfused and fixed with 2.5% glutaraldehyde. After decalcified in 10% EDTA, cochleas were post-fixed with 1% osmium tetroxide, dehydrated and embedded on aluminum stubs, coated with gold particles.

### Immunohistochemistry and synaptic counts

After washes with 0.1% Triton X-100 in PBS, sections on adhesion microscope slides were blocked with 10% normal goat serum (ZLI-9021, ZSGB-BIO) and incubated with rabbit anti-*SCN11A* polyclonal antibody (AT322395, 1:200, OriGene, Rockville, MD), guinea pig anti-Nav1.9 polyclonal antibody (AGP-030, 1:200, alomone labs, Israel), mouse anti-CtBP2 (612044, 1:100, BD Biosciences), in 10% goat serum diluted in 0.1 M PBS at 4 ℃ overnight and then incubated with secondary antibodies containing anti-mouse Alexa Fluo™ 488 (lot 1810918, 1:400, goat, Thermo Fisher), anti-rabbit Alexa Fluor™ 568 (lot 1494753, 1:400, goat, Thermo Fisher), or anti-guinea pig Alexa Fluro^TM^647 (A-21450, 1:400, goat, Thermo Fisher). Cell nuclei were labeled by DAPI.

For pre-synaptic ribbons counts, all pieces of each basilar membrane for each mouse were imaged with converted locations into frequency by ImageJ Plugin according to Cochlear Frequency Mapping in Whole Mounts (MASSACHUSETTS EYE AND EAR). Confocal z stacks from 4.0 to 64.0 kHz regions form each cochlea were taken using a LEICA DMi8 microscope equipped with 63× oil immersion lens. Five random fields at the region of 20–50% [mouse standardized cochleogram according to Müller et al. [22] from the apex were chosen for pre-synaptic ribbons counting, corresponding to the frequency around 8–16 kHz. The z stacks with 10 μm (0.75 μm step size) were set to ensure all the synaptic specializations were imaged. CtBP2 puncta in superimposed confocal z stacks was visualized and counted as presynaptic counts for each IHC. Each image usually contained 17–25 IHCs [23].

### Haematoxylin Eosin (H&E) staining and SGN counting

The sections were deparaffinized with xylene twice (10 min each), followed by re-hydration in 2 changes of absolute alcohol (5 min each), 95% alcohol, 85% alcohol, 75% alcohol (2 min each) and washed in distilled water briefly. Then the sections were stained in Harris hematoxylin solution and counterstained in eosin-phloxine solution. The sections were dehydrated through 95% alcohol and absolute alcohol and cleared in xylene twice, 5 min for each time. Finally, the mounted sections were observed under a light microscope [24].

The established method of paraffin slide was used for auditory neuron count [25]. In 5 mid-modiolus slices per cochlea from paraffin-embedding tissue, with 2 μm each on every 5th section, the perimeters of Rosenthal’s Canal were surveyed and the mean cell number of 5 slices was regarded as neuron count for one mouse. The number of SGNs with soma diameter equal or greater than 13 μm within the apex, middle and basal modiolus were chosen and counted manually with the assistant of Image J, respectively [26].

### Western blot analysis

Four wildtype mice and 3 Nav1.9 knockout mice were included in WB assay. Protein lysate from bilateral cochlea samples for each mouse was prepared in RIPA lysis buffer with the adding of complete protease inhibitor by Tissue Grinding Pestles, followed by keeping in an ice bath for 10 min. After centrifugation at 12,000 rpm for 10 min at 4 ℃, the supernatant mixed with loading buffer was denatured and loaded on a SDS-PAGE (12%) gel. Proteins were transferred to PVDF membrane for 90 min in an ice bath. After blocking, the membrane was incubated in primary antibodies of anti-Nav1.9 and anti-β actin with gentle agitation at 4 ℃ overnight, followed by HRP-conjugated secondary antibody incubation. Finally, the signal of HRP was detected using GE Healthcare’s ECL detection reagent. Antibodies in this study were as follows: polyclonal rabbit anti-SCN11A polyclonal antibody (AT322395, 1:1000, OriGene), beta Actin mouse monoclonal antibody (TA811000, 1:1000, OriGene), anti-rabbit HRP-linked IgG (7074, 1:4000, Cell Signaling Technology), goat anti-mouse HRP-linked IgG (H+L) (LK2003, 1:4000, sungene biotech).

### Auditory brainstem response (ABR) and ECochG recording

Mice were anesthetized using an intraperitoneal injection of pentobarbital sodium (50 mg/kg) and kept on a thermal insulation blanket. Click stimuli or pure tone stimuli from 2 to 16 kHz were generated by Tucker Davis Technologies System (TDT) and delivered by a MF1 speaker (TDT) [27]. The intensity of the tone stimuli was calibrated using a sound level meter with 1/4-inch pressure-field microphone (B&K). Response signals were recorded from the scalp vertex by needle electrodes, the postauricular region of the ipsilateral ear as reference, and the contralateral ear as ground. Auditory thresholds were determined as the lowest sound intensity with reproducible and recognizable waves by decreasing the sound intensity from 100 to 10 dB SPL in 5 dB steps. Mean ± SD was plotted as a function of stimulating sound frequencies, or a function of months after birth at one frequency for each genotype [28].

For ECochG recordings, the left cochlea was exposed through a dorsolateral posterior-auricular surgical approach. Once the bulla had been opened by a cutting burr, the recording electrode was placed on the round window membrane with the aid of a micromanipulator. The reference and ground electrodes were placed in the muscle near the cochlea. When the CAP of the auditory nerve was probed, the acoustical stimuli were generated by TDT system and delivery to a MF1 speaker. Cochlear amplification was achieved through an amplifier, averaged 1024 times. The CAPs were evoked at 16 kHz tone burst and threshold was obtained. The amplitude and latency of the first positive peak (P1) amplitude were measured. All experiments were carried out in a double-walled sound-attenuating room.

### Statistical analysis

Data was analyzed by SPSS version 17.0 software (SPSS Inc., Chicago, IL, USA) and plotted using GraphPad prism 7 (Graphpad, USA), and expressed as mean ± SD. Quantitative data statistical analysis was performed by one-way ANOVA followed by post-hoc tests (as appropriate) for multiple group comparisons, independent samples *t* test and nonparametric test (Mann–Whitney U test) for two-group data. *p < 0.05, **p < 0.01 and ***p < 0.001 were considered to indicate a statistically significance.

## Results

### Disruption of *SCN11A*

Nav1.9 KO mice were genotyped by PCR using genomic DNA from ear marginal tissue. Primer pair 1 and primer pair 2 was used to specifically distinguish WT and Nav1.9^−/−^ genotypes. The resulting products of WT, Nav1.9^+/−^ and Nav1.9^−/−^ were analyzed by agarose gel electrophoresis (Fig. 1b and Additional file 1: Fig. S4). The deletion of 347 bp, starting in exon 3 and ending in exon 5 of the coding sequence of *SCN11A* mRNA, induced a truncated form of Nav1.9 with 96 amino acid residues, and led to the reading frame shift mutation followed by a premature translational-termination codon (Fig. 1c). The mutation resulted in dysfunction of the protein with no membrane-spanning domain. No significant phenotypic difference was found in Nav1.9^−/−^ mice from WT littermates in their size, weight, coat color, locomotor activity, eating or drinking behavior, fertility and life span. Besides, necropsy and histology between Nav1.9^−/−^ and WT mice were considered indistinguishable, apart from a poor protein expression of Nav1.9 in the cochlea of Nav1.9^−/−^ mice (Fig. 1d and Additional file 1: Fig. S5).

### Nav1.9 is expressed in the inner ear and auditory pathway of wild-type mice

**Fig. 2:**
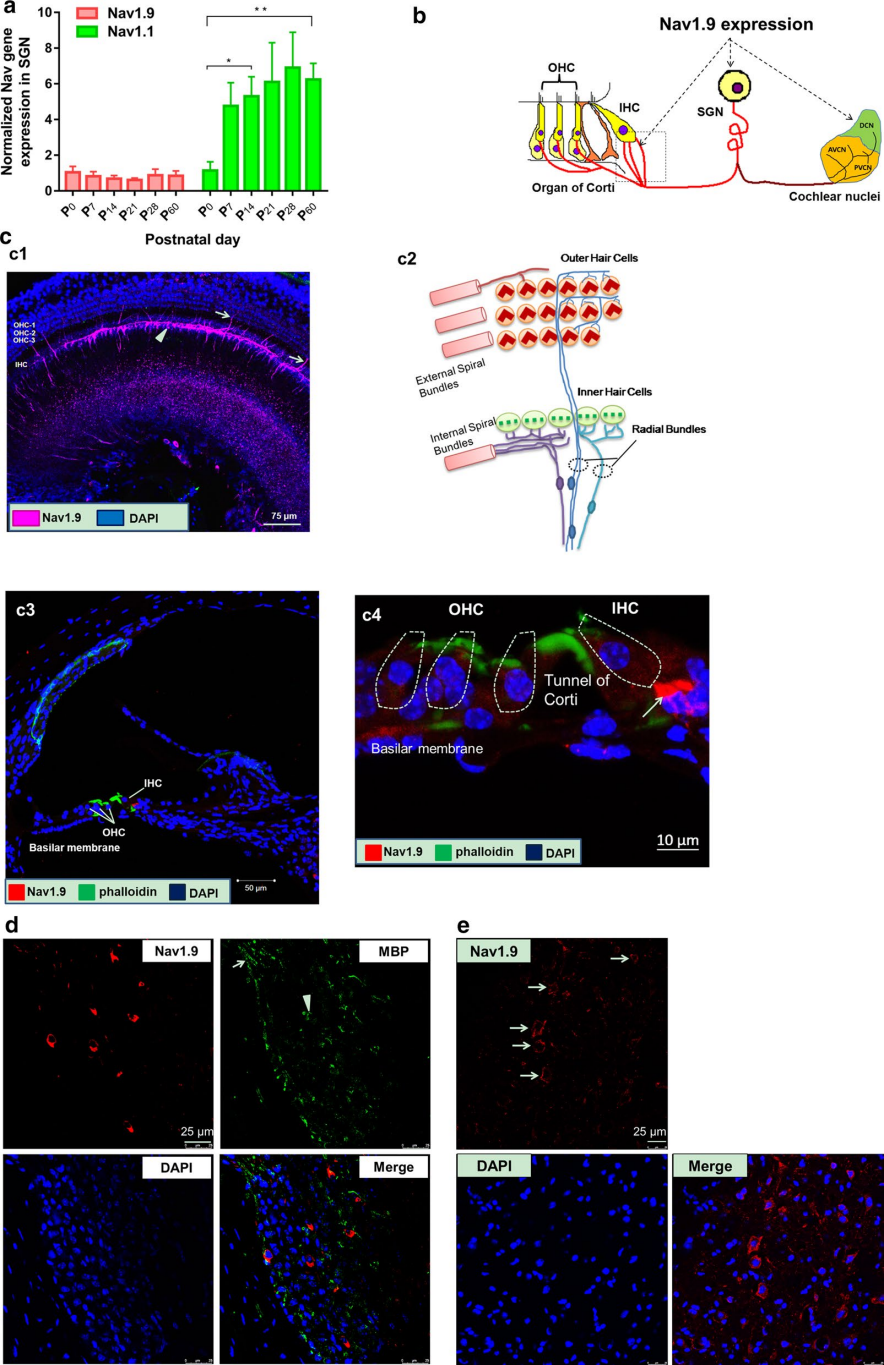
Distribution of Nav1.9 in primary auditory afferents. **a** The voltage-gated sodium channel Nav1.9 and Nav1.1 mRNA levels in modiolus of WT ICR mice at the postnatal 0, 7th, 14th, 21th, 28th and 60th day. Each time point contains 5 mice. *p = 0.028, **p = 0.004. **b** A schematic representing the localization of Nav1.9 channels at primary afferent peripheral nerve endings on hair cells in cochlea, in SGN somata, in the auditory nerve located within the modiolus, and in the cochlear nuclei. **c** Nav1.9 is present in cochlea basilar membrane by surface preparation technique and immunofluorescence staining in cryo-section. **c1** Horizontal section showing three rows of OHCs and one row of IHCs. In a linear distribution below the IHCs, Nav1.9 (purple) is in the afferent endings beneath the IHC bases. Also stained are the afferent radial fibers leading through the tunnel of Corti to their first hemi-nodes beneath the foramina nervosa. Scales = 75 μm. **c2** The diagram of the cochlea’s afferent innervations pattern. **c3** Nav1.9 is in the nerve endings of internal spiral fibers or radial fibers beneath IHC (red), the cilia of which exhibit phalloidin labeling (green). Scales = 50 μm. **c4** The high magnification image of c3. Scale = 10 μm. **d** The expression of Nav1.9 in the SGNs of P60 WT mouse was measured by immunofluorescence. Nav1.9, MBP, and cell nucleus are stained as red, green and blue, respectively. **e** Some neurons from the dorsal cochlear nucleus are labeled by Nav1.9 (red)

The expression of Nav1.1 and Nav1.9 mRNA from modiolus was examined using qPCR in WT mice from P0 to P60 (5 mice in each time point). During cochlea development, the expression of Nav1.9 showed no significant difference at P7, P14, P28 and P60 (Fig. 2a, one-way ANOVA, *F* = 1.673, *p* = 0.18). However, the relative expression levels of Nav1.1 mRNA at P14 and P60 were significantly higher than that at P0 (Fig. 2a, one-way ANOVA with Tamhane’s post-hoc test, *p = 0.028, **p = 0.004).

The localization of Nav1.9 in the cochlea was primary afferent and efferent endings in the organ of Corti, SGN somata and cochlear nucleus (Fig. 2b). The immunostaining for Nav1.9 can be seen in inner spiral fibers beneath the inner hair cells (IHCs), radial fibers innervating IHCs, tunnel crossing fibers and outer radial fibers (Fig. 2c, c1, c3 and c4), according to the diagram of the cochlea’s afferent innervations pattern from Ballenger’s Otorhinolaryngology 18 (Fig. 2c, c2). Besides, some of spiral ganglion neurons (SGNs) that couldn’t be stained by anti-MBP antibody (a myelin sheath marker), were immunolabeled for Nav1.9 (Fig. 2d and Additional file 1: Fig. S3). Futhermore, some of neurons within the cochlear nucleus exhibited expression of Nav1.9 on cell membranes at a relatively low density (Fig. 2e), which was consistent with data from previous studies [19].

### Nav1.9^−/−^ mice are deaf progressively at 16 kHz

**Fig. 3:**
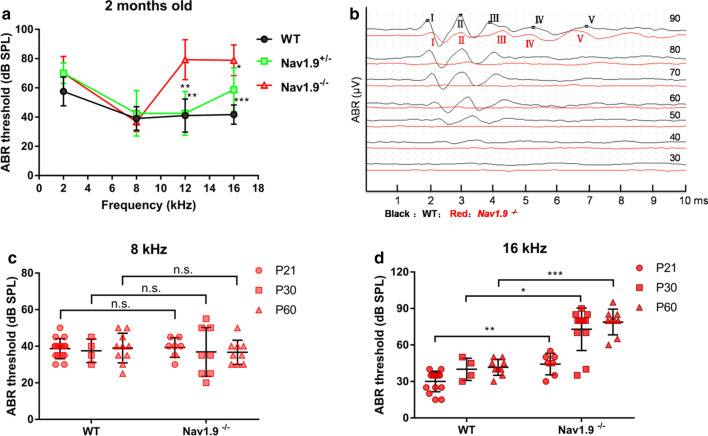
Audiological characterization of Nav1.9^−/−^ mice. **a** Mean ABR thresholds of six wild-type, four heterozygous and seven homozygous versus sound frequency, **p = 0.002, **p = 0.001 at 12 kHz compared with homozygous, *p = 0.01, ***p = 0.000 at 16 kHz compared with homozygous by one-way ANOVA with Bonferroni’s post-hoc test. **b** Example of ABR waveforms at 16 kHz in one ear of a wild-type superimposed on an example of ABR waveforms in one ear of Nav1.9^−/−^ mice. **c** ABR thresholds of WT and homozygous mice of postnatal day 21 to 60 at 8 kHz. *p* = 0.807 at P21; *p* = 0.932 at P30; *p* = 0.504 with independent samples *t* test; n.s.: not significant. **d** ABR threshold of WT and homozygous mice of postnatal day 21 to 60 at 16 kHz. *p = 0.016, **p = 0.006, ***p = 0.000 with Mann–Whitney test. Data are expressed as mean ± s.d

ABR was used to assess hearing thresholds in 2-month-old mice. The averaged ABR thresholds of Nav1.9^+/+^ mice, Nav1.9^±^ mice and Nav1.9^−/−^ mice showed no significant difference with each other at 2 kHz (Fig. 3a, one-way ANOVA, *F* = 2.954, *p* = 0.085) and 8 kHz (Fig. 3a, one-way ANOVA, *F* = 0.576, *p* = 0.571), respectively (Table 2). In addition, Nav1.9^−/−^ mice (n = 7) showed a remarkably higher average ABR threshold than Nav1.9^+/−^ (n = 4) and WT mice (n = 6) by 36.8 dB SPL (**p = 0.002) and 38.3 dB SPL (**p = 0.001) at 12 kHz (Fig. 3a, one-way ANOVA with Bonferroni’s post-hoc test), respectively. The average ABR threshold of Nav1.9^−/−^ mice was up to 78.9 ± 10.5 dB SPL, which is significantly higher than Nav1.9^±^ (58.8 ± 14.9 dB SPL) (*p = 0.01) and WT mice (41.7 ± 6.6 dB SPL) (***p = 0.000) at 16 kHz, while the average ABR threshold of Nav1.9^+/−^ was higher than WT mice (*p = 0.032) (Fig. 3a, one-way ANOVA with Bonferroni’s post-hoc test) as well. In all mice tested, threshold elevation correlated with reduced amplitudes of all ABR waves at 16 kHz were found in Nav1.9^−/−^ mice (Fig. 3b).

**Table 2:** ABR thresholds in Nav1.9^+/+^ (WT), ^+/−^, ^−/−^ mice at 2 months of age

Group	Frequency (kHz)			
	2	8	12	16
Nav1.9^+/+^	57.5 ± 9.9	39.0 ± 8.1	41.0 ± 11.4**	41.7 ± 6.6***
Nav1.9^+/−^	70.0 ± 7.1	42.5 ± 15.5	42.5 ± 15.0**	58.8 ± 14.9*
Nav1.9^−/−^	70.0 ± 11.5	36.7 ± 6.6	79.3 ± 13.7	78.9 ± 10.5

Asterisks indicate significant differences (*p < 0.05, **p < 0.01, ***p < 0.001) as compared with the Nav1.9^−/−^ group

To examine the onset time of hearing loss, ABR thresholds at the P21, P30, and P60 were measured at 8 and 16 kHz, respectively. At 8 kHz, the average ABR thresholds of WT mice was 38.7 ± 5.5 (n = 15) at P21 and 39.0 ± 8.1 dB SPL (n = 10) at P60. The ABR thresholds of Nav1.9^−/−^ mice was 39.3 ± 5.3 dB SPL (n = 7) at P21 and 36.7 ± 6.6 dB SPL (n = 9) at P60. There was no significant difference between the WT and KO mice (Fig. 3c, *t* = − 0.248, *p* = 0.807 at P21; *t* = 0.087, *p* = 0.932 at P30; *t* = 0.683*, p* = 0.504 at P60 with independent samples *t* test); At 16 kHz, the ABR threshold of WT mice was 30.0 ± 8.5 dB SPL (n = 15) at P21, which was significantly lower than their Nav1.9^−/−^ littermates of 44.3 ± 8.9 dB SPL (n = 7) (*U* = 14.5, *Z* = − 2.751, **p = 0.006 with Mann–Whitney U test). Besides, the Nav1.9^−/−^ mice showed a significant ABR threshold elevation compared with their WT littermates at P30 (72.9 ± 17.5 dB SPL (n = 12) vs. 40.0 ± 9.1 dB SPL (n = 4), *U* = 4.5, *Z* = − 2.406, *p = 0.016 with Mann–Whitney U test Fig. 3d), and at P60 (78.9 ± 10.5 dB SPL (n = 9) vs. 41.7 ± 6.6 dB SPL (n = 9), *U* = 0.000, *Z* = − 3.608, ***p = 0.000 with Mann–Whitney U test Fig. 3d). The ABR threshold shifts of Nav1.9^−/−^ mice in comparison with WT littermates, were 14.3 dB SPL at P21, 32.9 dB SPL at P30, and 37.2 dB SPL at P60, respectively, exhibiting progressive hearing loss (Table 3).

**Table 3:** ABR thresholds in Nav1.9^+/+, −/−^ mice at different age

Age (day)	8 K		16 K	
	WT	Nav1.9^−/−^	WT	Nav1.9^−/−^
P21	38.7 ± 5.5	39.3 ± 5.3	30.0 ± 8.5	44.3 ± 8.9**
P30	37.5 ± 6.5	36.9 ± 13.3	40.0 ± 9.1	72.9 ± 17.5*
P60	39.0 ± 8.1	36.7 ± 6.6	41.7 ± 6.6	78.9 ± 10.5***

Asterisks indicate significant differences (*p < 0.05, **p < 0.01, ***p < 0.001) as compared with WT group

### CAP recording

**Fig. 4:**
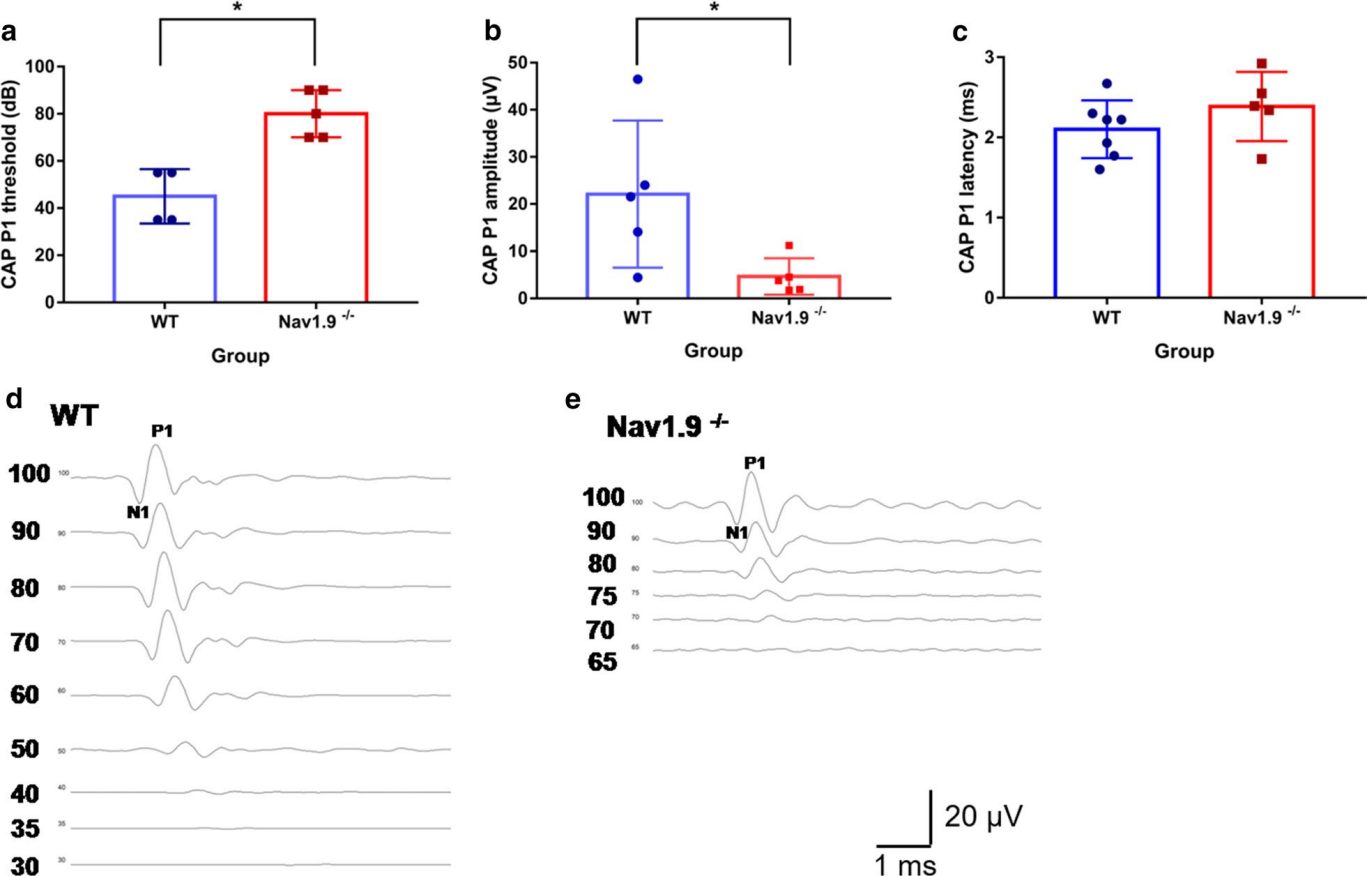
Auditory compound action potentials are affected by Nav1.9 knockout. Nav1.9 knockout induces higher CAP P1 threshold, *p = 0.013 with Mann–Whitney test (**a**), lower CAP P1 amplitude, *p = 0.041 with independent samples *t* test (**b**), compared with WT group; **c** the CAP P1 latency is not affected at the time point of postnatal day 60, *p* = 0.242 with independent samples *t* test. **d** Representative CAP waveforms from a WT mouse. **e** Representative CAP waveforms from a Nav1.9^−/−^ mice. Data are expressed as mean ± s.d

In order to identify the lesion region of hearing loss affected by Nav1.9, the CAP was recorded from the round window of cochlea. The average threshold of CAP of Nav1.9^−/−^ mice was 80.0 ± 10.0 dB (n = 5) which was significantly higher than the WT mice (45.0 ± 11.5 dB SPL, n = 4) (*U* = 0.000, *Z* = − 2.491, *p = 0.013 with Mann–Whitney U test, Fig. 4a). The average amplitude in P1 wave of Nav1.9^−/−^ mice at 80 dB SPL was 4.6 ± 3.9 μV (n = 5) which was significantly lower than that form the WT mice (22.1 ± 15.6 μV, n = 5), (*t* = 2.434, *p = 0.041, independent samples *t* test, Fig. 4b). In addition, the average latencies of P1 waves from WT and Nav1.9^−/−^ mice were 2.1 ± 0.4 ms (n = 7) and 2.4 ± 0.4 ms (n = 5), respectively, with no statistical difference (*t* = − 1.244, *p* = 0.242 with independent samples *t* test) (Fig. 4c). The representative CAP waveforms of WT and Nav1.9^−/−^ were shown in Fig. 4d, e.

### Ribbon synapse counting

**Fig. 5:**
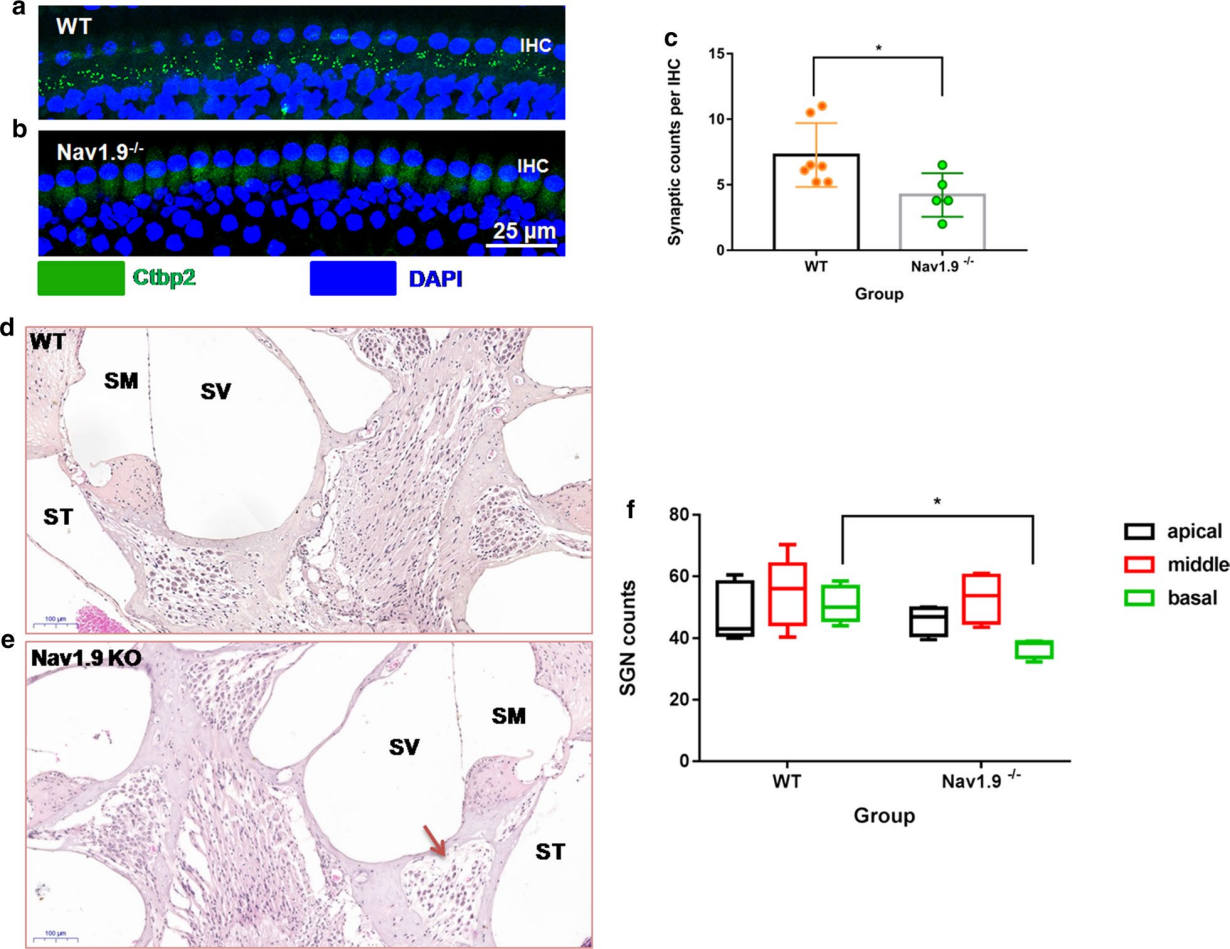
Nav1.9 knockout affects ribbon synapse density and survival of spiral ganglion neurons. Representative images of ribbon synapse immunostained with Ctbp2 (green) from WT (**a**) and Nav1.9^−/−^ mice (**b**). **c** Quantitative analysis of ribbon synapse counts per IHC from five randomly selected visual fields for each mouse. n = 7 for WT group, n = 5 for Nav1.9^−/−^ group. *p = 0.034 by Mann–Whitney test. Representative images of spiral ganglion neurons in Rosenthal’s canal from WT (**d**) and Nav1.9^−/−^ mice (**e**). **f** Spiral ganglion neuron counts in the basal turn, the middle turn and apical turn together in 3 midmodiolar sections for each animal. n = 5 for WT group, n = 4 for Nav1.9^−/−^ group. *p = 0.014 by Mann–Whitney test. Data are expressed as mean ± s.d

The quantitative changes in ribbon presynaptic RIBEYE were stained using antibodies against CtBP2. The average number of CtBP2 puncta in WT mice (n = 7) was 7.3 ± 2.4 per IHC, which was statistically higher than that in Nav1.9^−/−^ mice with 4.2 ± 1.6 per IHC (n = 5) (*U* = 4.5, *Z* = − 2.122, *p = 0.034, Mann–Whitney U test, Fig. 5a–c). This data indicates that Nav1.9 KO induced auditory deficits by a mechanism including cochlear synaptopathy.

### SGN counting

The number of spiral ganglion neurons (SGNs) was counted. The average number of the SGNs in the basal turn of cochlea of Nav1.9^−/−^ mice (n = 4) was 37.0 ± 3.1, which is significantly lower than that of WT mice (n = 5) with 51.0 ± 5.8 (*U* = 0.000, *Z* = -2.46, *p = 0.014, Mann–Whitney U test, Fig. 5d–f). However, the number of SGNs in both apical (*t* = 0.518, *p* = 0.622 with independent samples *t* test) and middle turn (*t* = 0.703, *p* = 0.823 with independent samples *t* test) showed no statistical difference between the two groups. The average numbers of SGN were 45.8 ± 4.7 in apex and 53.8 ± 8.2 in middle for Nav1.9^−/−^ mice; 48.3 ± 9.3 in apex and 54.6 ± 11.2 in middle for WT mice, which was in accordance with impaired temporal neural code at high frequency, such as 16 kHz.

### Nav1.9 knockout with intact OHCs

**Fig. 6:**
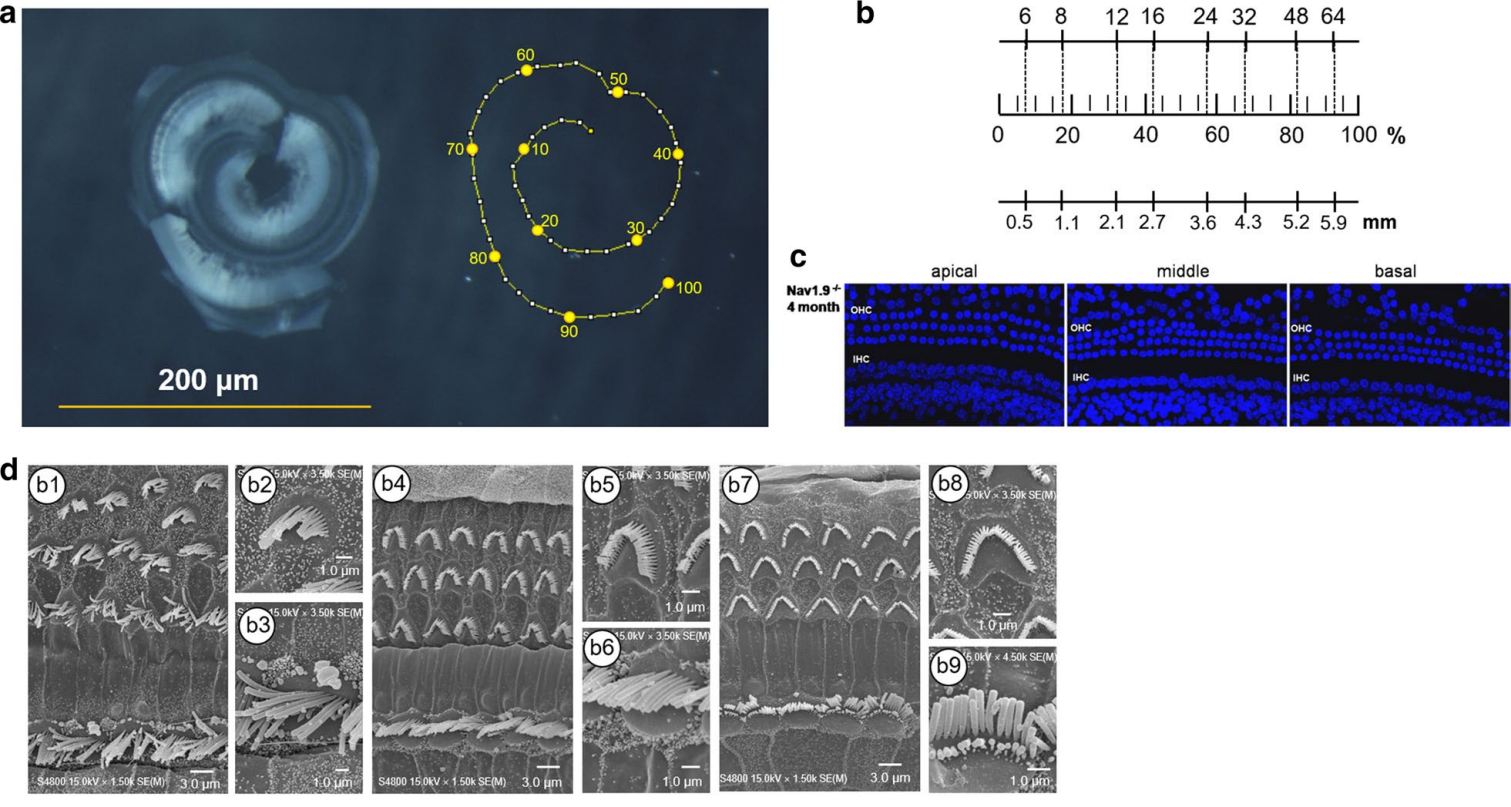
Nav1.9 knockout does not affect the morphology of hair cells. **a** The digital image of a dissected cochlea including the hook region (left) from a 4 months old mouse. Schematic drawing of the same cochlea with percent distance from the apex plotted (right). **b** Scale is showing frequency, percent distance from the apex, and distance (mm), according to Müller et al. [22]: x = 100 − (156.53 − 82.46 * log(F)). The full basilar membrane length is 6.3 mm for this particular cochlea. C, Images of organ of Cortis from 4 months old mice stained by DAPI (blue), with the apical turn (0–25% distance from the apex), the middle turn (30–55% distance from the apex) and the basal turn (60–85% distance from the apex). **d** Images of the organ of Corti of Nav1.9^−/−^ mice at postnatal ages of 2 months by SEM, containing the apical turn (**b1**–**b3**), the middle turn (**b4**–**b6**), and the basal turn (**b7**–**b9**)

Nav1.9^−/−^ mice had spared outer hair cells (OHCs) function of mechanoelectrical transduction and cochlear amplification. To explore the hair cell morphology in the region of ABR threshold shift, a standard cochleogram of a 4 months old Nav1.9^−/−^ mouse was provided (Fig. 6a), containing scales of frequency, percent distance from the apex according to Müller et al. [22] (Fig. 6b**)**. No significant hair cell missing from base to apex was found in cochleograms from DAPI staining, especially in the middle turn (30–55% distance from the apex) (Fig. 6c). Subsequently, we investigated the morphology of inner and outer hair cell stereocilia by scanning electron microscopy. Adult Nav1.9^−/−^ mice at 2 months had well-formed and evenly spaced stereocilia bundles, with an equal height within each row in the apical (Fig. 6d, b1–b3) middle (Fig. 6d, b4–b6), and basal turn as well (Fig. 6d, b7–b9).

## Discussion

This paper we have three novel findings on the expression and function of Nav1.9 in auditory system. (1) Nav1.9 was detected in the SGNs somatathat couldn’t be immunolabeled by MBP; (2) Nav1.9 KO mice showed a high frequency hearing loss at 2 months age. (3) Morphology experiments demonstrated reduced synapses and number of auditory nerves in the basal turn of the cochlea in Nav1.9 KO mice.

### Nav1.9 is predominantly present in peripheral auditory nerve

We found that Nav1.9 was located in nerve terminals in a plane beneath the IHCs. Also stained are the afferent radial fibers leading through the foramina nervosa (FN) (Additional file 1: Fig. S1), and a small part of SGNs somata, their peripheral and central initial segments. The distribution of Nav1.9 detected in the dorsal cochlear nucleus was consistent with a previous study [19]. In addition, Nav1.9 labeled fibers run longitudinally beneath the IHCs among scattered CtBP2 in the presynaptic membrane (Additional file 1: Fig. S2), suggesting that this sodium channel may play a role in modulating neurotransmitter such as glutamic acid in presynaptic membrane. This hypothesis was consistent with less heat-induced calcitonin gene-related peptide release form the skin in Nav1.9 KO mice. Alternatively, Nav1.9 may affect the ribbon synapse plasticity as well.

The distribution of Nav1.9 in auditory afferent nerve is similar to that in functionally identified nociceptors [29]. The localization of Nav1.9 channels at primary afferent peripheral nerve endings in the skin, is in analogy to that in afferent nerve ending beneath the IHCs; Nav1.9 in DRG somata is in analogy to that in SGN somata; Nav1.9 is expressed in central nerve endings in the dorsal horn of the spinal cord, in analogy to that in dorsal cochlear nucleus. However, more evidences that Nav1.9 distributes in auditory primary afferent are still needed.

Furthermore, as one of Nav channels specifically expressed in the peripheral nervous system, Nav1.9 may be expressed with unique patterns. Unlike Nav1.6 which is localized in the outer spiral fibers and their sensory endings beneath the OHCs, and Nav1.2 which is localized to the unmyelinated efferent axons and their endings on the IHCs and OHCs, Nav1.9 is predominantly present in the inner spiral fibers beneath the IHCs, which consist of distinctive neuronal plexus (glutamic acid decarboxylase (GAD)-positive fibers and gamma-aminobutyric (GABA)-positive fibers) intimately associated with IHCs [30]. Compared with mRNA expression of Nav1.1, which is a main contributor to auditory nerve spike generation [31], the expression of Nav1.9 mRNA was much lower at P7 at the onset of hearing. The different expression profile of Nav1.9 with Nav1.1, suggests that this channel may contribute less to the action potentials generation. Third, Nav1.9 KO mice showed elevated CAP thresholds compared to the WT mice. Moreover, the decreased amplitudes of CAP may result from loss of neural activity, as a result of a decrease in SGN count in Nav1.9 KO mice. Therefore Nav1.9 channels may serve as an amplifier of receptor potentials, facilitating neuronal excitation, rather than contributing to action potential generation.

### Nav1.9 knockout mice show reduced ribbon synapses

The average number of ribbon synapse in adult Nav1.9 KO mice was lower than their WT littermates. All synaptic ribbons contain a unique ribbon-specific scaffolding protein called RIBEYE, which contains a C-terminal B-domain that is identical with the transcription factor CtBP2 [32]. The reduced CtBP2 intensity in Nav1.9 KO mice suggests that Nav1.9 is essential for integrity of presynaptic nano-domains that position release-ready synaptic vesicles. An analogy to this hypothesis is the possible role of Nav1.9 in modulating neurotransmitter release in the dorsal horn of the spinal cord at the first synapse mediating pain signaling [29]. The mechanism of Nav1.9 in modulating morphology and function of ribbon synapse is still ambiguous. One candidate factor neurotrophin-3 (Ntf3) has been reported to regulate ribbon synapse density in the cochlea. Supporting cell-derived Ntf3 promotes ribbon synapse regeneration after acoustic trauma [33]. In the CCI model of neuropathic pain, Ntf3 is capable of attenuating expression of Nav1.9 mRNA and protein [34], indicating a possible ability of Ntf3 modulating the plasticity of ribbon synapse via Nav1.9 in cochlea.

Knockout of Nav1.9 induces postsynaptic degradation including loss of both ribbon synapse and SGNs, which may not affect the activity of OHCs. Although Nav1.9 distributes tunnel fibers to OHCs, knockout alleles doesn’t cause significant OHCs loss or dysfunction, due to the possible compensation of other sodium channels such as Nav1.2 or Nav1.6 tracking afferent innervation of OHCs [35].

### Nav1.9 knockout mice have progressive hearing loss in high frequency

This study showed Nav1.9 knockout mice with profound hearing loss at 16 kHz as early as 1 month after birth, while at 8 kHz no significant acoustic trauma was found from 21 days to 2 months after birth. Redox imbalance induced progressive haring loss was explored in a *Dusp1* deficient mouse model, which progressively trigger inflammation and apoptotic cell death [36]. It is implied that Nav1.9 knockout may trigger a stress imbalance. In support of this hypothesis, the current density of Nav1.9 was increased by inflammatory mediators, such as interleukin-1β [37], thus rendering DRG neurons hyperexcitable and leading to pain in inflammatory disorders.

Hearing loss in high frequency may result from reduced SGNs in the basal turn by affecting the cellular survival or development in Nav1.9 knockout mice. One explanation may be that the neurotrophins neurotrohin-3 (NT-3) and brain-derived neurotrophic factor (BDNF) could regulate the gradient expression of Nav1.9 in the organ of Corti. Both BDNF and NT-3 expressed in the cochlea support SGN survival during development. As NT-3 expression is highest in the cochlear apex and lowest in the base, and high expression on the modiolus side, the negative regulation might contribute to the higher expression level of Nav1.9 in the base. BDNF, the receptor tyrosine kinase (TrkB) and Nav1.9 has been reported as a gating mechanism in both hippocampal neurons in CNS and SH-SY5Y line [9]. Nav1.9 knockout may have significant effect on the survival of SGN in the base, through reduction of BDNF-TrkB-Nav1.9 pathway. The exact mechanism why Nav1.9 knockout affected SGN survival in the basal turn, rather than the middle and apex, is still unclear.

## Conclusions

For the first time, our study provides evidence that Nav1.9 is expressed in SGNs and essential for high frequency hearing in the mouse cochlea. Since transcriptome analysis shows that *SCN11A* is expressed in SGNs in neonatal and adult mice [38–40], it would be interesting to determine if Nav1.9 is expressed in type I or type II SGNs or both. It is also interesting to determine if Nav1.9 is related to the fibers with low and medium rates or high levels of spontaneous activity. Although transcriptome analysis shows that *SCN11A* is still expressed in adult hair cells [41, 42], we speculate that reduced number of presynaptic CtBP2 is secondary to loss of SGN terminals in the basal turn in Nav1.9 KO mice. But our study does suggest Nav1.9 function can affect the survival of SGN. The precise mechanism of Nav1.9 in modulating auditory neural function deserves further study.

## Supplementary Information

**Additional file 1: Figure S1.** The expression of Nav1.9 is located in spiral bundles beneath the IHCs bases. A, Horizontal section showing Nav1.9 (red) in the afferent endings and in the afferent radial fibers leading through the FN (arrow). B, Cross section showing Nav1.9 (red) labeling afferent radial fibers leading through the FN (arrow). T: the tunnel of Corti; PP: the phalangeal processes. **Figure S2.** Nav1.9 labeling spiral bundles run among scattered CtBP2 puncta in presynaptic membrane. A, Staining with DAPI in cochlea basilar membrane. B, Immunostaining with Nav1.9 (red), containing radial bundles (arrow) and suspected afferent (dashed arrow) from unmyelinated Type II ganglion cells cross the tunnel of Corti (T) to innervate OHCs. C, Immunostaining with CtBP2 (green) puncta beneath IHCs (arrowhead). D, Bright field of cochlea basilar membrane, showing one row of OHCs, the phalangeal processes (PP), and one row of IHCs. E, Image with overlapping fluorescent channels. Scale = 25 μm. **Figure S3.** Anti-MBP antibody labeling myelin sheath covering type I afferent and neuron soma (Green). BF: bright field. Immunostaining with MBP (green) in type I afferent fibre (arrow), and type I SGN soma (arrowhead) are shown, respectively. **Figure S4.** The genotype was identified by PCR. Line M: DL2000 DNA Marker; Line 1: the PCR product of tissue from WT mouse with Prime 2 (shortened to “WT with Primer 2”); Line 2: WT with Primer 1; Line 3: heterozygous with Primer 2; Line 4: heterozygous with Primer 1; Line 5: homozygous (female) with Primer 2; Line 6: homozygous (female) with Primer 1; Line 7: homozygous (male) with Primer 2; Line 8: homozygous (male) with Primer 1. **Figure S5.** The expression of Nav1.9 in the cochleas of Nav1.9^−/−^ mice (n = 3) or WT mice (n = 4) was measured by western blot.

## Data Availability

The datasets generated and/or analyzed during the current study are available from the corresponding author on reasonable request.

## Abbreviations

KO: Knockout
SGNs: Spiral ganglion neurons
ABR: Auditory brainstem response
ECochGs: Electrocochleogram recordings
IHCs: Inner hair cells
OHCs: Outer hair cells
WT: Wild-type
CAP: Compound action potential
SAPs: Single action potentials
DRG: Dorsal root ganglion
BDNF: Brain-derived neurotrophic factor
CMH: C mechano-heat-sensitive
*Atoh1*: Atonal homolog 1a
CWCs: Cartwheel cells
DCN: The dorsal cochlear nucleus
DSB: Double-strand break
gRNAs: Single-guide RNAs
FN: Foramina nervosa
