# Supplementary figures and images for: SCN11A gene deletion causes sensorineural hearing loss by impairing the ribbon synapses and auditory nerves
Source: BMC Neurosci. 2021 Mar 22;22:18. doi: 10.1186/s12868-021-00613-8 (PMC7986359; doi:10.1186/s12868-021-00613-8)

**Additional file 1**.

**Figure S1:**


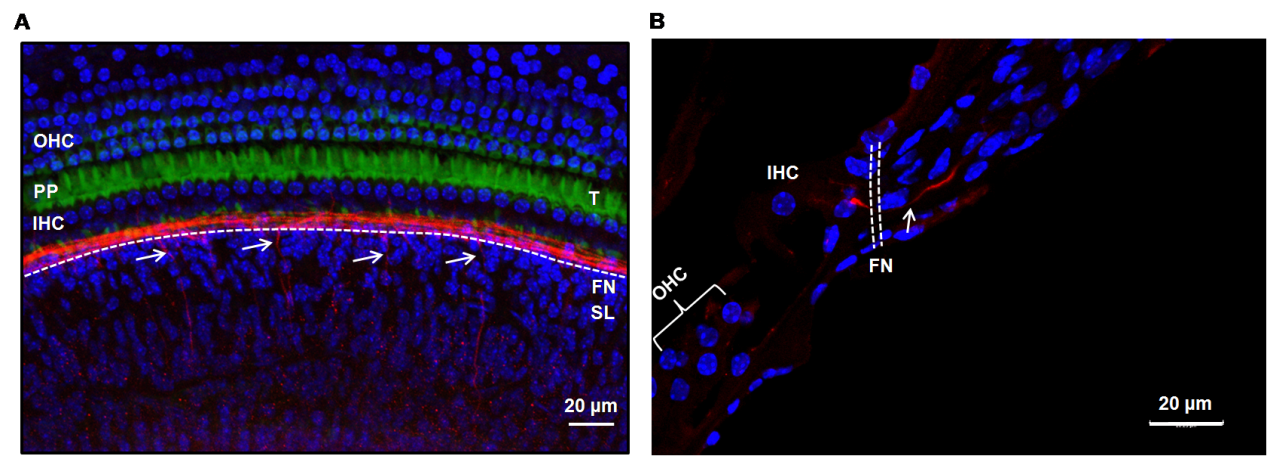


**Figure S2:**

**
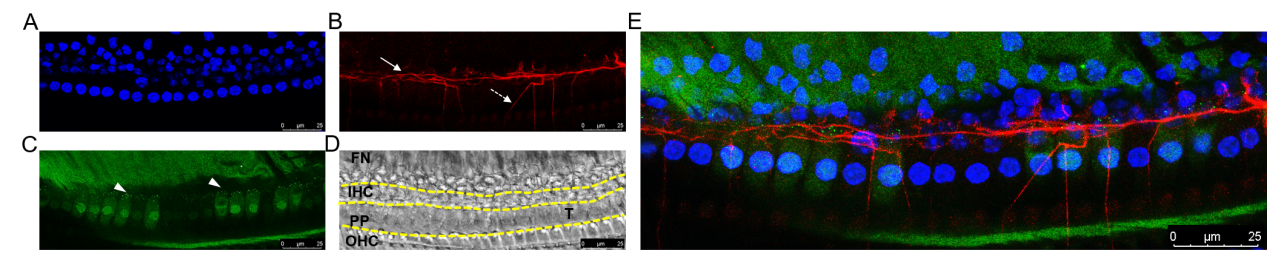
**

Figure S3:


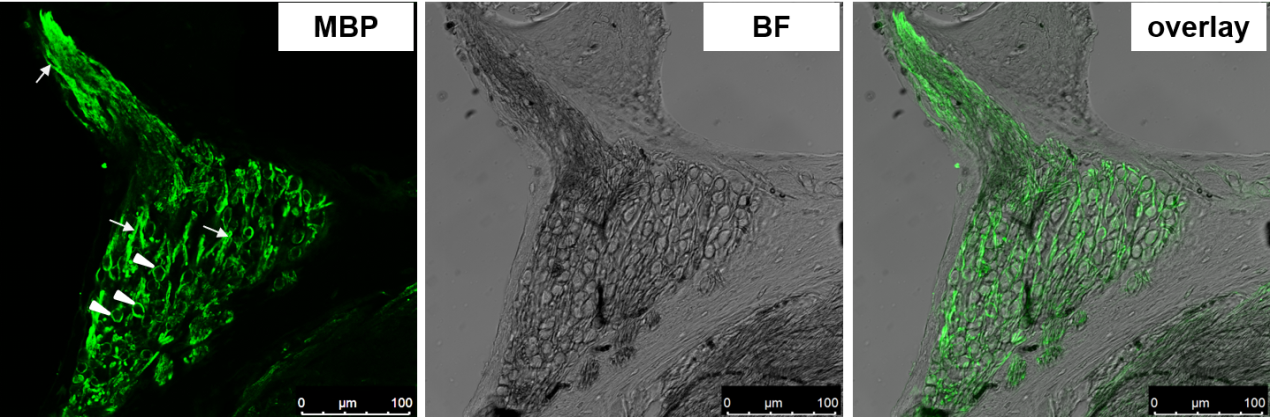


Figure S4:

**
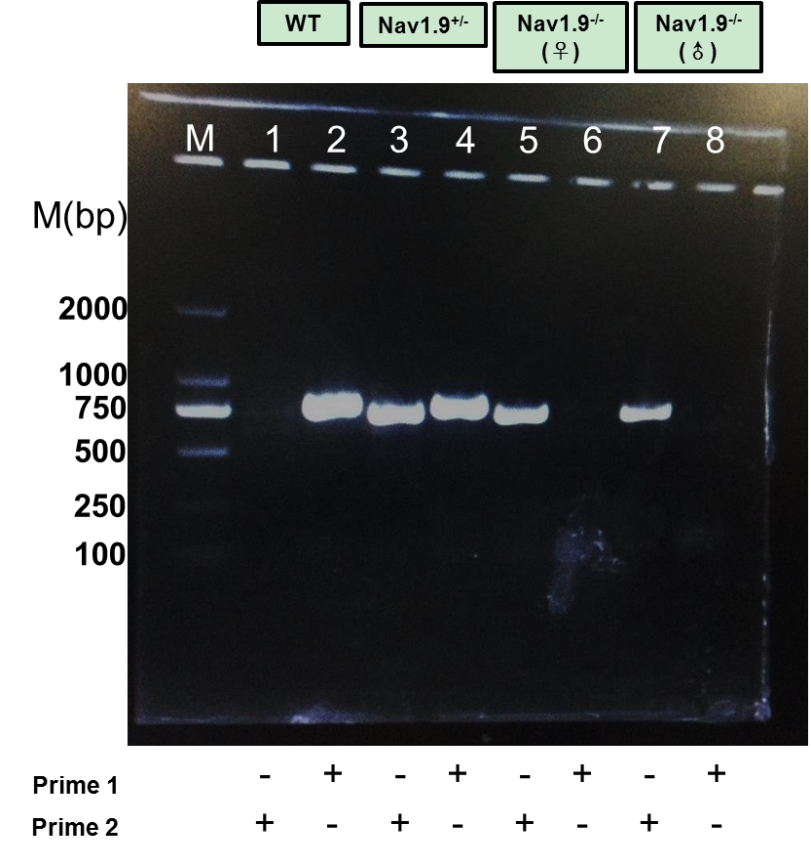
**

Figure S5:


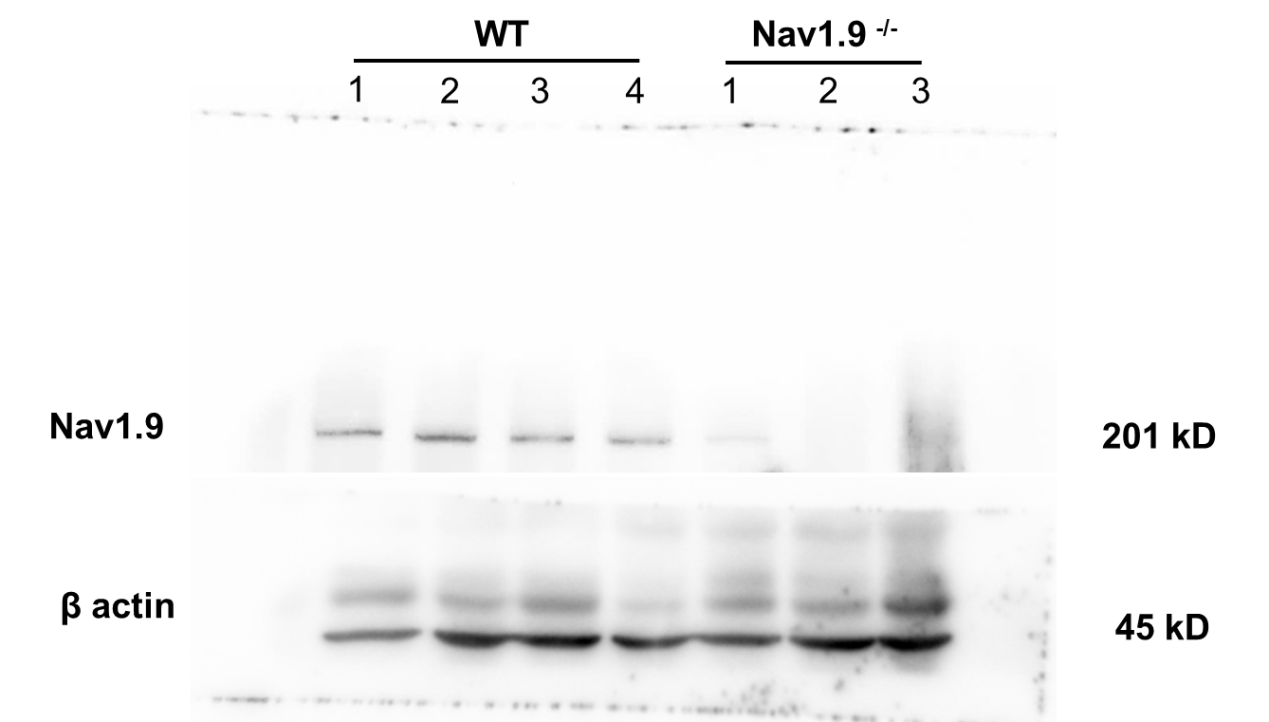

Supplement: Supplementary file 1 — Additional file 1: Figure S1. The expression of Nav1.9 is located in spiral bundles beneath the IHCs bases. A, Horizontal section showing Nav1.9 (red) in the afferent endings and in the afferent radial fibers leading through the FN (arrow). B, Cross section showing Nav1.9 (red) labeling afferent radial fibers leading through the FN (arrow). T: the tunnel of Corti; PP: the phalangeal processes. Figure S2. Nav1.9 labeling spiral bundles run among scattered CtBP2 puncta in presynaptic membrane. A, Staining with DAPI in cochlea basilar membrane. B, Immunostaining with Nav1.9 (red), containing radial bundles (arrow) and suspected afferent (dashed arrow) from unmyelinated Type II ganglion cells cross the tunnel of Corti (T) to innervate OHCs. C, Immunostaining with CtBP2 (green) puncta beneath IHCs (arrowhead). D, Bright field of cochlea basilar membrane, showing one row of OHCs, the phalangeal processes (PP), and one row of IHCs. E, Image with overlapping fluorescent channels. Scale = 25 μm. Figure S3. Anti-MBP antibody labeling myelin sheath covering type I afferent and neuron soma (Green). BF: bright field. Immunostaining with MBP (green) in type I afferent fibre (arrow), and type I SGN soma (arrowhead) are shown, respectively. Figure S4. The genotype was identified by PCR. Line M: DL2000 DNA Marker; Line 1: the PCR product of tissue from WT mouse with Prime 2 (shortened to “WT with Primer 2”); Line 2: WT with Primer 1; Line 3: heterozygous with Primer 2; Line 4: heterozygous with Primer 1; Line 5: homozygous (female) with Primer 2; Line 6: homozygous (female) with Primer 1; Line 7: homozygous (male) with Primer 2; Line 8: homozygous (male) with Primer 1. Figure S5. The expression of Nav1.9 in the cochleas of Nav1.9−/− mice (n = 3) or WT mice (n = 4) was measured by western blot. [file 12868_2021_613_MOESM1_ESM.docx]
